# Toxocarosis in a patient with autism spectrum disorder presenting with severe hypereosinophilia and acute respiratory distress: a case report

**DOI:** 10.1007/s00436-024-08119-y

**Published:** 2024-01-17

**Authors:** Daniel Üblagger, Herbert Auer, Milina Bezakova, Veronika Kirchlechner

**Affiliations:** 1 St. Anna Children’s Hospital, Department of Pediatrics, Medical University Vienna, Vienna, Austria; 2 Medical Parasitology, Institute of Specific Prophylaxis and Tropical Medicine, Center of Pathophysiology, Infectiology and Immunology, Medical University Vienna, Vienna, Austria

**Keywords:** Toxocarosis, Autism spectrum disorder, Geophagia, Albendazole

## Abstract

A boy with known autism spectrum disorder was transferred to our department due to a rapidly worsening respiratory situation. The patient’s history revealed previous treatment with albendazole against a *Toxocara* infection 2 weeks prior in Poland. Blood analysis showed such severe eosinophilia and markedly elevated levels of IgE that, initially, a hematologic malignancy was suspected. However, diagnostic workup including autoimmune diagnostic, molecular genetic testing, fluorescence in situ hybridization (FISH), bone marrow aspiration, and parasitological testing led to the diagnosis of an insufficiently treated *Toxocara* infection. Treatment with albendazole and prednisone (six cycles for 4 weeks each) was administered. This treatment regime led to prompt improvement of symptoms and normalization of laboratory findings.

## Introduction

Toxocarosis results from human infection with larvae of the ascarid nematodes *Toxocara canis* and *T. cati*. Humans serve as paratenic hosts and acquire the infection by ingesting *Toxocara eggs*, which contain infective third-stage larvae (Rostami et al. 2019). Contamination of parks, playgrounds, etc., with the feces of infected animals (dogs, cats, and other carnivores) is the main source of transmission. Children showing symptoms of pica are therefore at high risk. Consumption of raw or undercooked meat from other paratenic hosts like cattle, sheep, and chickens can be a further route of transmission. Once inside the small intestine, the larvae exit the eggs, penetrate the intestinal wall, and then reach the circulation, from where they migrate to the liver, lungs, and heart, leading to an inflammatory response and clinical symptoms depending on the affected organs (Ma et al. 2018). Depending on the clinical presentation, four clinical forms have been recognized: visceral larva migrans; ocular larva migrans; neurotoxocarosis; covert, common, and cardiac toxocarosis (Auer and Walochnik 2020). Visceral larva migrans can lead to pneumonia and hepatitis as the larvae migrate through the lungs and liver, respectively. Patients often present with wheezing, coughing, pruritic urticaria-like cutaneous lesions, myalgia, and eosinophilia. Additional symptoms may include lymphadenopathy, myocarditis, arthritis, and nephritis (Taylor et al. 1988). Ocular larva migrans can lead to blindness due to severe vitritis, tractional retinal detachment, or cystoid macular detachment. It is caused by larval localization in the eye and the resulting immunological response (Pivetti-Pezzi 2009). Neurotoxocarosis results from the migration of larvae in the central nervous system, causing meningitis and encephalitis, and commonly presents with non-specific symptoms such as fever or headache (Finsterer and Auer 2013). Generally, the majority of patients infected with *Toxocara* do not develop severe systemic manifestations. More frequently, they develop non-specific symptoms including fever, nausea, vomiting, abdominal pain, hepatomegaly, pulmonary symptoms, lymphadenitis, limb pain, lethargy, and behavioral disorders. Based on the results of a case–control study, Taylor et al. described in children the aforementioned clinical entity as “covert toxocarosis” (Taylor et al. 1987). A similar syndrome in adults was outlined by Glickman et al. and was subsequently called “common toxocarosis.” Thus, “covert” and “common” toxocarosis seem to represent the same clinical entity depending on whether a child or an adult is suffering from it (Glickman et al. 1987). Worldwide, toxocarosis is one of the most commonly documented zoonotic helminth infections. Generally, human toxocarosis is more prevalent in rural areas than urban ones. Seroprevalence in Europe spans from 2.4 to 31% (Strube et al. 2020).

Diagnosis is based on clinical presentation and imaging findings as well as results from serological tests, in particular enzyme-linked immunosorbent assays (ELISA) for *T. canis* excretory or secretory (TES) antigens, which are then confirmed via immunoblot (Ma et al. 2018).

## Case description

**Fig. 1:**
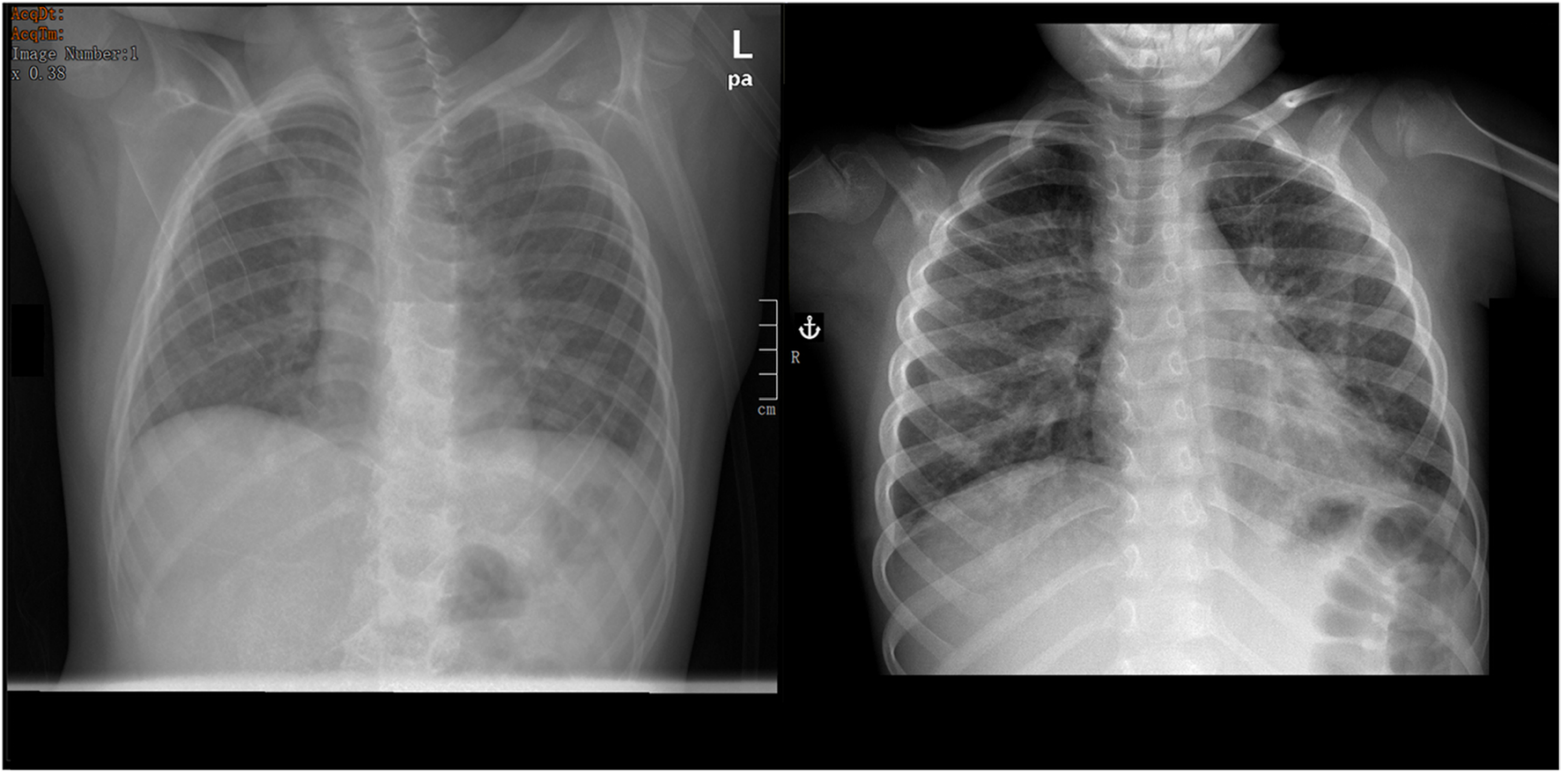
Chest X-ray showing migratory infiltrates indicative of eosinophilic infiltration

A 6-year-old boy with known autism spectrum disorder was admitted to our hospital due to marked respiratory distress and subfebrile temperatures. When the boy was admitted, the parents reported that they often took walks in the forest and that the boy suffered from geophagia. Furthermore, the parents disclosed that for the last couple of weeks, they had noticed some behavioral changes, including increased restlessness and aggression in the boy. Additionally, they outlined that 2 weeks ago, the boy had been admitted to a pediatric department in Poland, where he had been diagnosed with *Toxocara canis* infection and received treatment with albendazole for 5 days. On admission, the boy was hypoxemic, with an oxygen saturation of 86% on ambient air, and tachypneic. On lung auscultation, coarse rhonchi and wheezes were present. The remaining clinical examination was unremarkable. The full blood count showed remarkable leukocytosis (88,750/μL) with severe eosinophilia (63,010/μL) and marked hypergammaglobulinemia with total IgE of 14,776 IU/mL. Electrolytes, creatinine, and liver function parameters were normal. Chest radiographs showed a migratory pulmonary infiltrate indicative of Loeffler’s syndrome (Fig. 1).

The autoimmune workup, including anti-nuclear (ANAs) and anti-neutrophil cytoplasmic antibodies (ANCAs), was negative. Molecular genetic testing of the peripheral blood showed neither the Janus kinase (JAK) 2 V617F point mutation, nor the FIP1-like1-platelet-derived growth factor receptor (FIP1L1-PDGFRA) fusion gene, nor the D816V mutation in the tyrosine kinase KIT. Additionally, fluorescence in situ hybridization (FISH) did not reveal any immunoglobulin heavy chain (IGH) rearrangements. Flow cytometry did not show leukemic cells in the peripheral blood. The bone marrow aspirate showed increased cell infiltration with eosinophiles of all stages of maturation without any lymphoblasts.

Serologically, no signs of a viral infection with HIV were detected. Serological tests came back negative for ascariasis, echinococcosis, fasciolosis, strongyloidosis, trichinellosis, and cysticercosis but highly positive for toxocarosis. Enzyme-linked immunosorbent assay (ELISA) detecting human IgG antibodies to *Toxocara* excretory/secretory antigens displayed > 100 antibody units (AU), indicating a significant increase over the antibody units shown in previous serological testing in Poland. Serological testing was then confirmed via Western blot. Based on the marked eosinophilia and the very high antibody level against *Toxocara* antigens, the diagnosis of a still-ongoing disease was made. Echocardiography, electrocardiography, and cranial magnetic resonance imaging (MRI) did not show signs of cardiac or cerebral involvement. Ultrasound of the abdomen and ophthalmoscopy were normal.

**Fig. 2:**
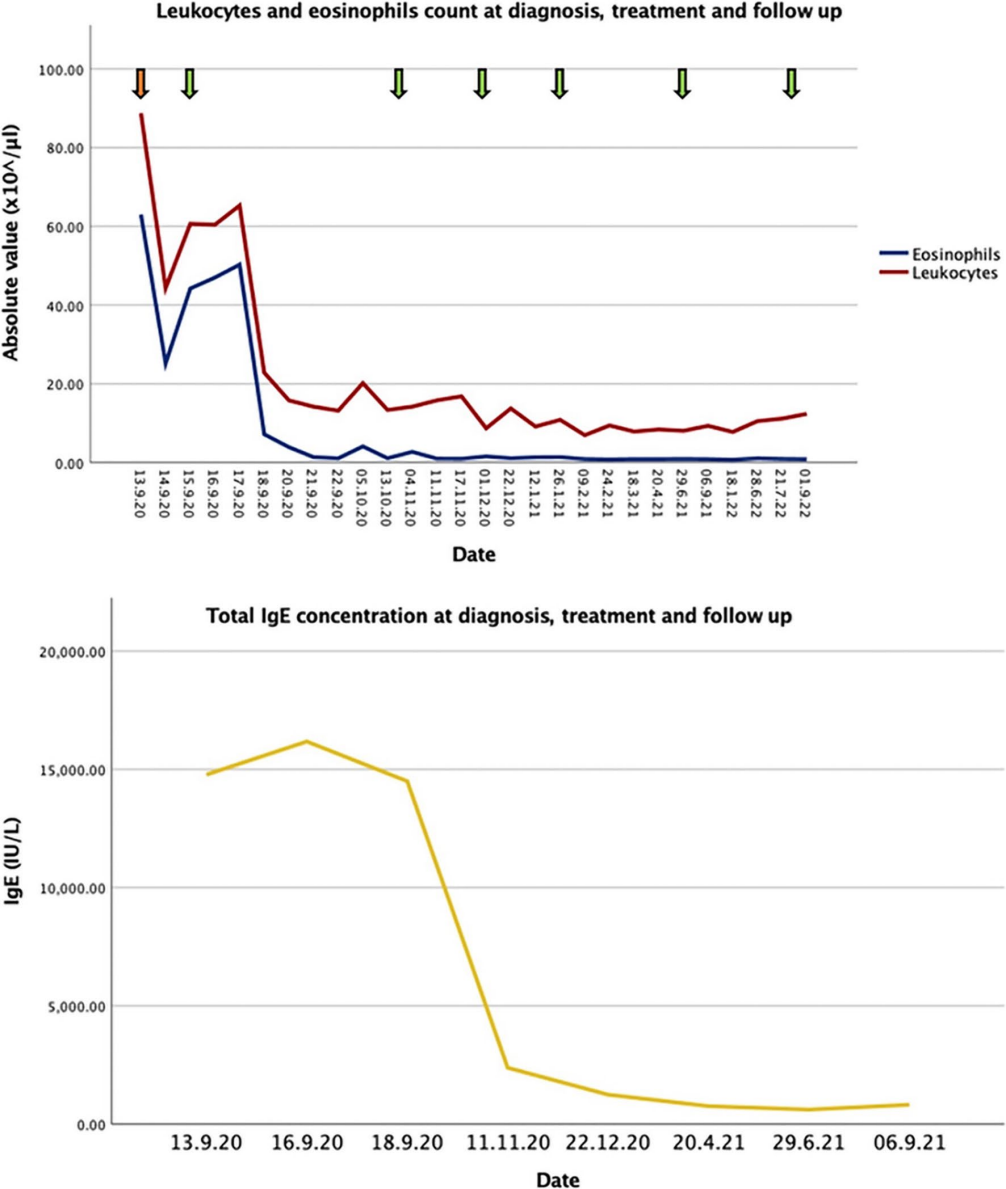
Leukocytes, eosinophils, and total IgE at the time of diagnosis, during treatment, and at follow-up

Due to the boy’s rapidly worsening respiratory situation, highly frequent inhalations with salbutamol and a continuous intravenous terbutaline bypass were started, and a constant oxygen supply was provided. Additionally, a single dose of systemic corticosteroids (prednisone 1 mg/kg body weight) was administered, leading to a stabilization of the boy’s respiratory situation, allowing him to be sedated in order to take a bone marrow aspirate. After establishing the diagnosis of toxocarosis, treatment with albendazole (7.5 mg/kg orally twice a day) and prednisone (2 mg/kg/day orally) was established. Since the 5-day treatment cycle with albendazole previously carried out in Poland did not lead to a satisfactory clearance of the infection and due to the severe eosinophilia and acute illness, the boy received four treatment cycles of albendazole (7.5 mg/kg orally twice a day), with each cycle lasting 4 weeks and a 2-week break in between cycles. The boy tolerated the treatment well. Besides food cravings from the corticosteroids, he did not suffer any side effects. He showed rapid improvements in his clinical symptoms (disappearance of tachypnoea and the coughing and no need for continuous oxygen supply, salbutamol inhalations, and terbutaline administration) and in the laboratory findings (normalization of the eosinophils count and IgE concentration) (Fig. 2).

Systemic corticosteroid therapy was tapered and finally terminated with fourth cycle of albendazole. Follow-up was done by repeated clinical examination and blood tests, which indicated no signs of respiratory symptoms and normal leukocyte counts. Despite normal leukocyte counts, the parents reported increasing restlessness and behavioral disturbances apart from the patient’s autism spectrum disorder. Thus, he received two additional treatment cycles with albendazole, resulting in the resolution of the symptoms. Although repeated ELISA tests for *Toxocara* remained highly positive with > 100 antibody units, at the last checkup, the parents outlined the patient’s good general condition after finishing the sixth treatment cycle with albendazole.

## Discussion

Pica is a known challenging behavior in patients with autism spectrum disorder (Matson et al. 2013). Our patient’s parents also reported that he suffered from geophagia, which put him at a higher risk of infection with *Toxocara* (Wiśniewska-Ligier et al. 2012). Therefore, the already-acquired *Toxocara* infection was in line with his increased risk due to his underlying condition. He had already received treatment against toxocarosis consisting of albendazole (7.5 mg/kg twice a day) for 5 days according to common recommendations. Yet, these recommendations are based primarily on clinicians’ experience, since randomized trials to determine the length of treatment are missing (Woodhall and Fiore 2014). Since our patient had already received treatment and due to the severity of his eosinophilia, a parasitic infection as the cause of his hypereosinophilia was not suspected at the beginning. Other etiologies for eosinophilia, including malignancies, autoimmune diseases, other infections, and hypereosinophilic syndrome, were ruled out step by step (Curtis and Ogbogu 2016). After all the performed tests revealed unremarkable results and chest radiography suggested pulmonary infiltration of eosinophils, serologically tests for parasitic infections were repeated, and due to the highly positive results of the *Toxocara* antibody tests, the diagnosis of insufficiently treated, still-ongoing *Toxocara* disease was established. In conclusion, with a seroprevalence of up to 31%, *Toxocara* infections are seen frequently in clinical practice. However, our patient’s severity of infection and acuteness of symptoms are rarely, and thus, a hematologic malignancy was initially suspected. It is, therefore, imperative to include helminth infections in the differential diagnosis of eosinophilia, even in severe cases. Helminths can induce severe eosinophilia, particularly when migrating through parenchymal organs. This is well illustrated in a case report of Kan H, which presents a boy experiencing a level of eosinophilia comparable to that of our patient, resulting from an infection with *Paragonimus westermani* (Kan et al. 1995).

Furthermore, this case impressively demonstrated that the commonly recommended treatment length of 5 days with albendazole might not be enough to sufficiently clear the parasitic infection. In a clinical trial, Stürchler et. al also showed that only 32% of toxocarosis patients were cured after 5 days of albendazole administration (Stürchler et al. 1989). Similar results were shown by Yoshikawa (Yoshikawa 2009). Thus, in Japan, the recommended treatment period for visceral larva migrans syndrome (VLM) caused by *Toxocara* spp. as well as by *Ascaris suum* larvae is one cycle of 4 weeks accompanied by a 2-week interval without therapy and then an additional cycle of 4 weeks of albendazole therapy (Hombu et al. 2019). Our therapy regimen was oriented toward the latter recommendation. However, due to the severity of the infection, the duration of the therapy was prolonged even further, and the flareup of behavioral symptoms impressively showed the need for the extension of therapy.

Noteworthily, the need to monitoring potential side effects, such as elevated liver values, during albendazole treatment, should be emphasized. Regular monitoring of liver function parameters is imperative in this regard. In case of significant elevation of these parameters, it might be necessary to pause therapy. Fortunately, our patient tolerated the treatment well, experiencing no significant side effects. Hence, the therapy could be administered without any interruptions.

The severity of our patient’s toxocarosis was most probably due to his kwon geophagia, which resulted in repeated ingestion of infective larvae, leading to a severe immunological response. The fact that ELISA tests still yielded highly positive results could either be because to anthelmintic treatment led to the disintegration of *Toxocara* larvae, resulting in a strong antigenic stimulation, or because the treatment did not kill all *Toxocara* larvae, resulting in ongoing antigenic stimulation by viable larva. The renewed flareup of symptoms consistent with the behavioral symptoms the boy experienced prior to the first treatment cycle and appropriate to covert toxocarosis supports the latter hypothesis. However, due to his pica disorder, he also could have ingested new *Toxocara* eggs, leading to the new symptoms.

Regardless, it is essential that our patient has regular clinical checkups to monitor for new symptoms, check for an increase in leukocyte and eosinophil counts, and initiate a new treatment cycle of albendazole if necessary.

## Data Availability

Not applicable.
